# Supplementary material for: What influences engagement with a bipolar disorder self-management app? A qualitative investigation of use of the PolarUs app
Source: PLOS Digit Health. 2025 Oct 10;4(10):e0001017. doi: 10.1371/journal.pdig.0001017 (PMC12513578; doi:10.1371/journal.pdig.0001017)
Supplement: S2 File — (DOCX) [file pdig.0001017.s002.docx]

**Supplementary File 2.**

**Qualitative Interview Guide**

**Introduction:**

Hello ___, this is ____ from CREST.BD. How are you?

The focus of this interview is to learn about your experiences - good or bad - of using the PolarUs app. The first questions I will ask you for feedback on specific aspects of the app, and your experiences of it compared to any other health apps you have used. The second set of questions will focus on what it was like to use the app in the context of your daily life. The final set of questions will be about what you learned through using the app, and whether you made any changes in your life as a result of what you learned.

At the end of the interview, you’ll have time to add any of your thoughts that you think are important to our research but that weren’t discussed. The interview will take about 45 minutes.

As you know, I’ll be audio recording this interview. [specific to zoom interviews: “This recording will be audio only – I won’t be recording video.”] I’m going to start recording now. After we transcribe the interview, the audio file will be deleted, and your name and identifiers, like your location, will be removed during transcription. After the interview, you'll receive a gift certificate to thank you for your time. Do you have any questions before we begin?

**First set: App feedback**

What were your expectations of the PolarUs app?

Can you tell me about your experiences using the PolarUs app?

Are there any particular aspects of the app that you liked? How about disliked?

How did the PolarUs app compare to any other health apps you have used (now or in the past)?

**Second set: Engagement**

Research tells us that’s it’s not always easy to use an app on a regular basis. Were there any aspects of the app design that encouraged you to use it on a regular basis? Were there any other factors which facilitated your use of the PolarUs app?

- Probe: If yes, what were they?

Were there times when you found it difficult to use the app as often as recommended?

- Probe: If yes, what happened?

Did anything happen that made it possible for you to pick up the app again?

Were there any times where bipolar disorder symptoms had an impact on your use of the PolarUs app?

**Final set: Behaviour change and quality of life**

Did you learn anything new from using the PolarUs app?

Did you put into practice any of the strategies that you selected?

- Probe: If changes were made, which ones? How/ what enabled you to do so?
- Probe: If no changes made, why not? What were the barriers?

Since you started using the PolarUs app, have you noticed any changes in your quality of life?

- Probe: If yes, how so?
- Probe: If no, why not?

Did you share any materials from the PolarUs app or talk about it with others?

What was it like to monitor your QoL using the weekly and monthly check-in surveys?

**Conclusion:**

Thank you so much for your time today. I just have a few final questions.

If you could change or add anything to this app, what would it be?

If you were given the opportunity, would you continue using this app? Why/why not?

Would you recommend this app to somebody else with bipolar disorder? Why/why not?

Is there anything else you would like to share with me or comment on?

End with a thank you for their time and sharing their insights about their experiences.
