# Supplementary material for: What influences engagement with a bipolar disorder self-management app? A qualitative investigation of use of the PolarUs app
Source: PLOS Digit Health. 2025 Oct 10;4(10):e0001017. doi: 10.1371/journal.pdig.0001017 (PMC12513578; doi:10.1371/journal.pdig.0001017)
Supplement: Attachment — Submitted filename: Response to reviewers.docx [file pdig.0001017.s004.docx]

| **Reviewer** | **Review comment** | **Our response** |
| --- | --- | --- |
| Editor | Please amend your detailed Financial Disclosure statement. This is published with the article. It must therefore be completed in full sentences and contain the exact wording you wish to be published.  1. Please clarify all sources of funding (financial or material support) for your study. List the grants (with grant number) or organizations (with url) that supported your study, including funding received from your institution.  2. State the initials, alongside each funding source, of each author to receive each grant.  3. State what role the funders took in the study. If the funders had no role in your study, please state: “The funders had no role in study design, data collection and analysis, decision to publish, or preparation of the manuscript.”  4. If any authors received a salary from any of your funders, please state which authors and which funders. | The format of this statement has been amended to align with journal requirements. |
|  | Please send a completed 'Competing Interests' statement, including any COIs declared by your co-authors. If you have no competing interests to declare, please state "The authors have declared that no competing interests exist". Otherwise please declare all competing interests beginning with the statement "I have read the journal's policy and the authors of this manuscript have the following competing interests:" | The format of this statement has been amended to align with journal requirements. |
|  | We note that you have indicated that there are restrictions to data sharing for this study. For studies involving human research participant data or other sensitive data, we encourage authors to share de-identified or anonymized data. However, when data cannot be publicly shared for ethical reasons, we allow authors to make their data sets available upon request. For information on unacceptable data access restrictions, please see <http://journals.plos.org/plosone/s/data-availability#loc-unacceptable-data-access-restrictions>.    Before we proceed with your manuscript, please address the following prompts:    a) If there are ethical or legal restrictions on sharing a de-identified data set, please explain them in detail (e.g., data contain potentially identifying or sensitive patient information, data are owned by a third-party organization, etc.) and who has imposed them (e.g., a Research Ethics Committee or Institutional Review Board, etc.). Please also provide contact information for a data access committee, ethics committee, or other institutional body to which data requests may be sent. | As this is a qualitative study, the data set involves richly detailed interviews where individuals spoke about their life circumstances and experiences. Even if potentially identifying details like names, ages, and locations were removed from the data set, it is still possible that these interviews, when viewed in full, contain enough information to identify the participants.  We note that participants in the qualitative study did not consent to sharing this data beyond the research team.  The study was approved by the University of British Columbia Behavioural Research Ethics Board. Contact details are as follows:  **UBC BREB Office** #102, Technology Enterprise Facility (TEF) III 6190 Agronomy Road   Vancouver, BC V6T 1Z3 Fax: 604-822-5093  [allison.stewartgomes@ubc.ca](mailto:allison.stewartgomes@ubc.ca) |
|  | We have noticed that you have uploaded Supporting Information files, but you have not included a list of legends. Please add a full list of legends for your Supporting Information files after the references list. | This has been added. |
| #1 | Thank you for sharing this manuscript. I found the detailed qualitative analysis interesting and thorough, it sets a good standard for MH app intervention evaluation. | Thank you for this positive feedback |
|  | Line 81: Where you reference 'symptoms', which ones? Additionally, for the prevalence statistics - are these global or US based? | We have added a line to clarify that self-management apps may have potential to reduce or prevent mood symptoms.  We have added text to clarify that these studies surveyed groups in the US, UK, and internationally. |
|  | Line 131: I would like to see some explanation for the range in interview times either here or in the limitations. Additionally, in the limitations you refer to waiting until the conclusion of the study for the interviews, but is there a reason some of them were so long after this (again, a wide range here). | One author (EM) was solely responsible for scheduling and conducting participant interviews. As such, the time between the conclusion of quantitative data collection and the qualitative data collection was impacted by scheduling conflicts. Other contributors to the delay were the fact that several participants completed their final interview in early-mid December, and both the interviewer and many participants were unavailable over the Christmas/Holiday period. We have clarified the timeframe in which interviews occurred in the Results, and have further commented on the implications of and rationale for this delay in the Limitations section.  Length of interviews was impacted by the depth with which participants responded to interview questions (including requests for elaboration). Our estimated sample size was based on previous qualitative interviews where similar variation was observed (interviews ranged from 20 to 70 min in length (M = 39.4 min, SD = 11.2) (Michalak et al., 2019; Morton et al., 2017). This information-power informed approach to sampling is further discussed below in our response to Reviewer 2. |
|  | Method - really great to see member checking of the thematic analysis! | Thank you for very much your positive feedback on this use of member checking. |
|  | Line 474: I think 'lover' is a typo of 'lower' | Thank you very much for bringing this to our attention! |
|  | I thought this finding was interesting 'Some participants avoided smartphone use during periods where they were recovering from mood elevation.' - it suggests perhaps some associated issues with encouraging online/device engagement in order to use the app, when this could be potentially harmful or interrupt recovery. This should be considered in the discussion or limitations. | We agree that this finding points to a need for clinicians and researchers to consider the potential downsides of app engagement, particularly in periods where mood elevation is a concern. We had commented on the risks of specific app features leading to overuse and mood elevation, and have added a comment linking this risk to findings reported in our paper.  We have also added a paragraph to the discussion expanding on suggestions for clinicians to support app engagement, focused specifically on changes to engagement with mood symptoms. In this paragraph, we recognised that while higher engagement has been seen as important for improving clinical outcomes in unipolar depression, a more cautious approach may need to be adopted for people living with bipolar disorder. |
| #2 | This study presents the reasons for engagement and disengagement with a bipolar disorder self-management app in a clear and methodologically correct manner. The qualitative focus on engagement provides some new and relevant insights for the development of mental health apps. Both research question and design are clearly stated and explained. The chosen qualitative interviews and their analysis are clearly stated and suitable for the research question. | Thank you very much for this positive feedback and recognition of the importance of this topic. |
|  | Sampling and data collection are clearly described and adequate by using a purposive subsample of participants. What is missing here is a rationale or justification for the chosen sample size of 48 invited patients. | In our published protocol (Michalak et al., 2022), we mentioned aiming to recruit approximately 30 participants, noting recommendations that qualitative sample size is informed by information power – that is, attention to a number of dimensions related to the research aims, informed by pragmatic considerations and the researcher’s own experiences (Malterud et al., 2016). When considering our proposed sample size, we considered our prior experiences of using qualitative methods to explore the use of digital health tools for BD (Michalak et al., 2019; Morton et al., 2017) – as the study aim was narrower and the sample was more specific as compared to our previous evaluation (which recruited 43 individuals to comment on four intervention arms), we considered a smaller sample size to be appropriate.  We also noted in the protocol that, based on information power guidance, “in practice the appropriateness of the sample must be evaluated during the research process.” During recruitment, we reevaluated the sample size in discussions between coinvestigators with respect to the diversity of experiences and demographic backgrounds. Although we reached out to individuals who reported more infrequent app use or diverse backgrounds, not all accepted the invitation to participate. To avoid further weighting the sample towards high engagers from already over-represented demographics (i.e., White women) we concluded recruitment once a sample size of 25 was achieved. This decision was also influenced by reflections on information power, as the quality of dialogue was strong, with the majority of participants offering rich and detailed responses to interview questions.  48 invitations were sent in order to reach the final number: 23 invitations were not responded to, or interview scheduling was unsuccessful. This process has been further elaborated upon in the methods section. |
|  | The data analysis process is described in sufficient detail. Strategies to enhance credibility are incorporated by member checking done by individuals with BD experience. | We appreciate this comment on the credibility of the analytic approach. |
|  | The findings are clearly presented and organized by meaningful and justified themes. However, the comparison of the results with existing literature is lacking; instead the Technology Acceptance Model is used as a comparison which is not sufficient. | Thank you for your comment, and we once again appreciate the positive feedback about the meaningfulness of the themes. Although we were aiming for a succinct discussion, we can see that introducing discussion of additional models of engagement would add richness to the consideration of existing literature. To expand on this discussion, we have also included a comparison with two other models that may be used to explain app engagement, including the Capability, Opportunity, Motivation and Behaviour model, and the Theoretical Domains Framework. We have also referenced a review paper which grouped existing literature on app engagement according to these frameworks for comparison. |
|  | Limitations of the study and research contributions are clearly stated and justified. | Once again, thank you for your positive feedback. |
| #3 | This qualitative study investigated the factors that influenced individuals’ engagement with a bipolar disorder (BD) self-management app. It is an interesting study within the journal scope. However, the research question is not clear, and more details regarding the methods and results should be provided. My detailed comments are as follows. | Thank you for this recognition of the relevance of the study topic. We hope to address your comments below. |
|  | Guidelines and criteria have been developed to ensure the validity and reliability of qualitative research and its analysis. Please ensure that the present qualitative research and its analysis follow these methodology guidelines and criteria. Also, please let readers know about the specific guidelines and criteria that are used. I have considered rejecting this submission due to the unclear validity and reliability of the qualitative analysis. However, I would like to hear what the authors have to say and will evaluate the paper again next time. If the paper still suffers from validity and reliability issues, it will be rejected. | We appreciate and agree with this reviewer’s emphasis on documenting the quality of our analysis. We note that concepts like validity and reliability, which may be used to support the quality of a quantitative analysis, do not transfer to qualitative analysis, which is underpinned by different research paradigms. Instead, concepts such as trustworthiness and rigor are more typically applied in qualitative research. We had previously commented on methodological decisions that were used to support the rigor of the analysis, including member checking and peer debriefing (which were commented on positively by Reviewers 1 & 2). To address this feedback, we have highlighted the specific criteria used (Lincoln and Guba, 1985) and provided more detailed examples of how our methodological decisions mapped onto these standards of credibility, transferability, dependability, and confirmability. |
|  | Abstract  The authors mentioned that they identified the app features associated with the drivers of engagement; however, I was unable to locate these results in the main text. Please organize the findings to improve the clarity. | We have modified this statement to clarify that description of app features is presented with the summary of each theme describing a driver of engagement.  This is further reiterated in the summary of results, where it is stated: “This includes participant perceptions of app features and content, and their experience of tasks associated with the intervention.” It would not be possible to separate out these descriptions within the themes without needing to repeat information. For example, for the theme summary of salience, we have contrasted how this experience varied in response to different app features and content. |
|  | Introduction The research question is not clear. It appears that the authors investigated the factors that influenced engagement with the app, as well as the factors that interacted with drivers of engagement. Please specify if there were two research questions and present them clearly. | Our broad, overarching research aim was to explore and describe prominent aspects of the experience of engaging with the PolarUs app. To address this comment, we have elaborated that this included consideration of self-reported patterns of engagement over time, as well as factors (both app features and other salient influences) that were perceived by interviewees to impact their engagement with the app. |
|  | Methods  Please provide more details and figures to illustrate the PolarUs App, as the features are unclear. Although the authors mentioned that details can be found in the protocol, the references to “three life domains” and “4 self-management strategies” are confusing in this manuscript. | To address this comment, we have provided additional description of the PolarUs intervention, and have included some screenshots to highlight key features (QoL psychoeducation and self-assessment). |
|  | In addition to the interview questions, the authors examined quantitative outcomes such as QoL.BD (both 12-item and 56-item versions). However, the results of these health-related outcomes are missing. The authors should provide the results of the descriptive analysis on these outcomes. In addition, they can examine whether the improvement in health condition can influence engagement with the app. | We appreciate this reviewer’s interest in the quantitative outcomes of the overarching PolarUs trial. The analyses that they are enquiring after are reported in the primary outcomes paper (currently under review), which used linear mixed modelling was to test the hypothesis that QoL (primary outcome) would improve over the intervention period and explore secondary outcomes (i.e., mood symptoms, self-efficacy, subjective recovery, self-compassion). Hierarchical cluster analysis was also used to investigate associations between app adherence and primary outcomes. The existence of this related manuscript was disclosed during the submission process; findings from these papers do not overlap with the present manuscript, which describes an analysis of qualitative interviews focused on experiences of engagement and disengagement. PLOS One guidelines are that unavailable and unpublished work, including manuscripts that have been submitted but not yet accepted (e.g., “unpublished work,” “data not shown”), are not cited in the reference list. Should the primary outcomes paper be accepted for publication before copy-editing of this manuscript, we will request that this reference be updated. |
|  | It is unclear why the interviews were conducted between 9 and 52 days following the conclusion of the intervention. This broad range might influence the participants’ responses to the interview questions. It is also noted that the mean duration was 28 days, which raised some concerns. The authors need to explain why these interviews were not conducted immediately after the completion of intervention. | We had addressed the influence of staffing and scheduling difficulties above in our response to reviewer 1, and have elaborated on the possible recall bias this introduces in the Limitations section. |
|  | For each of the resulting factors (or outcomes), how many participants mentioned it? Please provide a number for each. Along the same lines as the above comment, for “Several participants noted difficulty finding times where they were consistently available to complete their check-in …”, how many is “several”? This comment would have been addressed after addressing the above comment. I see the same issue in other places in the manuscript. Please fix all of them. | We appreciate this reviewer’s interest in the number of participants and comments which were integrated into the description of each theme. We note that (similar to our reply to this reviewer’s comment on reliability and validity) that as qualitative and quantitative methodologies are influenced by different research paradigms, it is not appropriate to apply the same quality standards of one tradition to the other. In fact, Braun and Clarke, the developers of the thematic analysis approach used in this paper (and commonly in qualitative psychology research more broadly) have argued against the use of numbers to characterise themes (Braun & Clarke, 2024), as have other qualitative researchers (e.g., Pyett, 2003). In brief, they suggest that numerically characterising themes may result in misinterpretation of findings. Participant/code counts may convey a positivist impression that a particular topic is important by virtue of how often it was mentioned – while this may be one contributor, frequency alone does not determine whether particular participant experiences should be highlighted. In fact, some strategies used to demonstrate rigour (e.g., negative case sampling) involve describing infrequent or contradictory responses in order to enhance the depth of theme descriptions. Presentation of numbers may also inappropriately lead readers to draw inferences about statistical generalisability, which is not the aim of qualitative research.  Further, unlike surveys, where participants are presented with a standard set of options to each question, participants in a qualitative interview can respond very differently even when presented with the same interview questions. Braun and Clarke therefore note that due to the nature of qualitative data collection, it cannot be presupposed that individuals who did not mention a particular idea did not agree – rather, it may simply be that they have not discussed it due to the flexible nature of qualitative interviewing. A numerical description, e.g., “X% of participants mentioned motivation”, may inaccurately convey the impression that only this group believed this to be important. It also does not represent the variation within a group who may discuss motivation, potentially collapsing brief mentions with more elaborative responses, or ambivalent and highly emphatic responses.  Finally, we note that inclusion of participant/code counts is not mentioned as a quality criterion for qualitative research (Lincoln & Gupa, 1985). In sum, we appreciate the reviewer’s interest in the quality of reporting, and hope the additional detail we have provided regarding how methodological decisions map to widely accepted quality criteria goes towards addressing this concern. |
|  | The authors did not specify how to complete daily and weekly check-in. Did they require the participants to log in to the app every day and answer some questions to check in? How did they calculate the percentage of completion? Did they conduct monthly check-in? | Thank you for noting this oversight – we have clarified in the methods section that daily check-in refers to the daily self-report mood and sleep items responded to in-app, and weekly check-in refers to the weekly collection of brief QoL.BD data in-app. The percentage of completion was calculated as a proportion of total possible check-ins during the study period. Monthly check-ins were not used to guide participant sampling as this was a three-month study, and this broad measure was therefore less informative with respect to behavioural engagement with the PolarUs app. |
|  | The authors identified three factors that influenced engagement with the app, including motivations, salience, and perceived effort. However, I am concerned whether these factors were derived from existing models or frameworks. Please provide more evidence to support their validity. | This qualitative investigation took an inductive approach, in which the themes generated are guided by participant responses, rather than pre-conceived theories or ideas. The inductive approach was selected for this study given it is capable or representing the unique contexts and experiences of participants, and it allows the research to remain open to new ideas that may not have been apparent when data is viewed through the lens of an existing framework. The inductive approach can be contrasted with deductive approaches, in which pre-existing theories and framework are used to structure and interpret the data (e.g., developing a coding framework based on constructs specified in established models). While the deductive approach can be used to test the relevance of existing theories, given the dearth of existing literature on engagement with mHealth interventions in people with bipolar disorder, we opted for the more exploratory, inductive approach. Although we previously specified use of an inductive approach in the Methods section, we have further elaborated on the definition and implications of this.  As previously stated, we did not set out to validate these themes against the data, and we are not proposing a model to replace any of the frameworks mentioned in the discussion. Rather, we seek to contribute to an ongoing discussion in the literature of aspects that should be considered when conceptualising or measuring engagement in the context of a particular population, by highlighting the subjective experiences of an understudied sample. We have modified the discussion to further emphasise this point. |
|  | Discussion  Since the authors mentioned the Technology Acceptance Model (TAM), it would be good to know why they did not conduct quantitative analysis related to TAM (e.g., path analysis). Such analysis would provide more important findings regarding the engagement with the BD app. | We appreciate this reviewer’s interest in further analyses that could provide quantitative data related to the themes identified in the present qualitative analysis. However, we note that the aim of the present study was not to *quantitatively* measure constructs related to TAM, but to *qualitatively* examine what are prominent aspects of the experience of engaging with the PolarUs app from the participant perspective, through data generated by individual interviews. It would not be appropriate to subject this kind of data to the statistical analysis proposed by this reviewer. We expect that this qualitative analysis can guide future quantitative studies of the PolarUs app, using larger sample sizes that would be appropriate for SEM. We have added a comment to the discussion suggesting that such a statistical analysis would be a potential future research direction that extends on the present findings. |

**References**

Braun V, Clarke V. Answers to frequently asked questions about thematic analysis. University of Auckland. Accessed November 19, 2024. <https://www.thematicanalysis.net/faqs/>

Braun V, Clarke V. Successful qualitative research: A practical guide for beginners. 2013. London:SAGE.

Malterud, K., Siersma, V. D., & Guassora, A. D. (2016). Sample Size in Qualitative Interview Studies: Guided by Information Power. *Qual Health Res*, *26*(13), 1753-1760. <https://doi.org/10.1177/1049732315617444>

Michalak, E. E., Barnes, S. J., Morton, E., O'Brien, H. L., Murray, G., Hole, R., & Meyer, D. (2022). Supporting Self-management and Quality of Life in Bipolar Disorder With the PolarUs App (Alpha): Protocol for a Mixed Methods Study. *JMIR Res Protoc*, *11*(8), e36213. <https://doi.org/10.2196/36213>

Michalak, E. E., Morton, E., Barnes, S. J., Hole, R., Crest.Bd, & Murray, G. (2019). Supporting Self-Management in Bipolar Disorder: Mixed-Methods Knowledge Translation Study. *JMIR mental health*, *6*(4), e13493-e13493. <https://doi.org/10.2196/13493>

Morton, E., Michalak, E. E., Hole, R., Buzwell, S., & Murray, G. (2017). *‘Taking back the reins’ – A qualitative study of the meaning and experience of self-management in bipolar disorder* (Vol. 228). <https://doi.org/10.1016/j.jad.2017.12.018>

Pyett, P. M. (2003). [Validation of Qualitative Research in the “Real World”](http://qhr.sagepub.com/content/13/8/1170). Qualitative Health Research, 13(8), 1170-1179.
