# Supplementary material for: What influences engagement with a bipolar disorder self-management app? A qualitative investigation of use of the PolarUs app
Source: PLOS Digit Health. 2025 Oct 10;4(10):e0001017. doi: 10.1371/journal.pdig.0001017 (PMC12513578; doi:10.1371/journal.pdig.0001017)
Supplement: Attachment — Submitted filename: Response_to_reviewers_auresp_2.docx [file pdig.0001017.s005.docx]

| **Reviewer** | **Review comment** | **Our response** |
| --- | --- | --- |
| Editor | The possibility of recall bias is significant. I can imagine that participants might not fully and accurately recall their experiences after 28.3 days (average) or 52 days (longest) of using an app, particularly if they disengaged early. Simply noting this as a study limitation does not address the issue, as recall bias remains present and could impact the results. | With respect to the reviewer and editor’s concerns about recall bias, we acknowledge that the time elapsed since the final clinical assessment may have impacted participant’s ability to remember details about the intervention and their use of it. However, we would argue that the impact of recall bias is more significant in papers that seek to specifically quantify an effect or association (e.g., reporting dietary intake, medication adherence, use of services), where systematic under or over-reporting can result in erroneous statistical conclusions being drawn. Here, we did not ask participants to characterise the duration or intensity of their use of the PolarUs app, but to identify factors that may have contributed to this. Our findings are not a conclusive statement on what factors determine engagement, but could be used to guide the collection and interpretation of relevant quantitative data in future, large scale evaluations of app engagement.  We have added the following text to the manuscript limitations:  “We further note that the optimal recall period for self-report surveys is suggested to be 6 months or less,^3^ as such, even for participants who disengaged use of the PolarUs app early, the timeframe of our interviews would still be well within recommendations. Finally, the time elapsed since the intervention in our analysis also contributed to our ability to find that the research context was a unique contributor to patterns of engagement with the PolarUs app, as some participants commented on the difference in their use post-trial as compared to when the study was ongoing. In light of ongoing discussions about how best to measure engagement with mental health apps, and concerns regarding the potential for the study environment to positively bias engagement as compared to real-world contexts,^1,2^ we argue that despite the potential of impacts to recall ability, these interviews represent an important contribution to the broader mHealth literature.”  Moreover, we wish to note that this period of time elapsed is not inconsistent with other papers recently published in PLOS Digital Health. For instance, a paper with a similar period of time elapsed collected qualitative survey data from participants who had a particular appointment type between 17^th^ January and 9^th^ April 2022, with data collected up to the end of May 2025.^4^ Other papers report significantly longer delays. One qualitative paper published in 2024 described interviews conducted between 2019 and August 2020, with participants who had called or registered a service between August 2017 and December 2018.^5^ Other examples include a paper published in 2025, exploring use of telehealth in the COVID-19 pandemic (specifically, during social distancing policies between April-May 2020), in which interviews were conducted between July and December 2022, a delay of over two years.^6^ Another paper described a survey during June 2022 regarding experiences of a digital health intervention in 2021.^7^ Despite the time elapsed, these papers offer important insights about technology use that can inform future research. |
|  | I agree with the reviewer that data saturation should be considered when determining when to stop including additional participants.  I also agree with the reviewer that adhering to a qualitative research reporting guideline is important. You can refer to the following resources: <https://jamanetwork.com/journals/jamasurgery/article-abstract/2778475> <https://academic.oup.com/intqhc/article-abstract/19/6/349/1791966> | With respect to concerns highlighted regarding saturation, we would like to re-iterate that qualitative research is a highly heterogenous family of methods. This has presented challenges for defining a core set of reporting guidelines. While the reviewer has raised concerns that would be fair to apply to specific qualitative traditions such as content analysis, their relevance for thematic analysis specifically has been challenged by the developers of this method Braun and Clarke. COREQ, despite adopting a specifically positivist perspective to qualitative research, is frequently misapplied to qualitative traditions that adopt a phenomenological or critical-realist orientation.^8^ For example, although COREQ calls for data saturation to be used, this implies a positivist perspective on data (i.e., there are a limited amount of themes to be discovered). This does not mesh with the assumptions of thematic analysis, which would posit that decisions about when to stop collecting data are inherently subjective (and should therefore be subject to the same type of reflexivity and rigour as other analytic decisions undertaken using this method). Furthermore, the failure to generate new themes (a common criteria used to determine saturation) does not safeguard against thin, poorly detailed, or irrelevant responses: technically, saturation could be quickly reached by interviewing a sample with limited experience or interest in the topic, or by using poor interviewing techniques (i.e., closed questions, limited probing). Information power is increasingly encouraged as a flexible method to reflexively consider the richness and relevance of data obtained to inform sample size. A detailed rebuttal against the usefulness of saturation as a concept for thematic analysis is outline by Braun and Clarke.^9^  Notably, the article linked to in the journal’s decision email^10^ itself notes “Given the variety of study types and methodological traditions represented within qualitative research, the SRQR and COREQ guidelines best serve their intended aim of improving qualitative study reporting when used as reminders for reporting rather than prescriptive requirements.” We also note that the SRQR was developed as an attempt to address limitations in COREQ discussed above, regarding “the need for flexibility in application to different approaches and the distinction between study rigor and reporting rigor. Indeed, the SRQR calls its standards recommendations, not criteria.”  We have therefore added the following text to the methods:  “The analysis is reported according to the Standards for Reporting Qualitative Research Framework (SRQR), in addition to recommendations to ensure quality practice in thematic analysis. A copy of the SRQR checklist is available in Supplementary File 1.”  “As the positivist assumptions of data saturation are not coherent with the reflexive nature of thematic analysis and do not safeguard against thin or underdeveloped themes, our final determination of sample size was guided by information power.”  We note that qualitative papers published in PLOS Digital Health have not universally used the COREQ recommend saturation-based approaches to determining sample size. Our approach to sampling, informed by information power, is consistent with several recently published papers in PLOS Digital Health.^11-13^ |
| #3 | I appreciate the authors’ efforts in revising the manuscript. Some of my comments have been well addressed; however, there are still methodological issues. In general, this manuscript is not yet ready for publication. | We appreciate the positive feedback on comments that were well-addressed in the previous round of revisions, and hope to provide further clarification regarding the acceptability of the methodological decisions made in this paper. |
|  | - The initial sample size was 30 in the published protocol, while it was reduced to 25 in the current study. The authors concluded recruitment once a sample size of 25 was achieved, which seemed quite casual. They provided justifications that they aimed to “avoid further weighting the same sample towards high engagers from already over-represented demographics (i.e., White woman)”; however, this can be problematic: 1) The issue of over-represented demographics regarding gender and ethnicity can influence the interpretation of the findings. The authors need to discuss the potential limitations. 2) It is unclear whether the other demographics were also over-represented. Stratification by demographics would be recommended. 3) The authors used purposive sampling to promote diversity in the sample, while this method might have introduced selection bias and limited generalizability of the findings. | In the initial published protocol, we provided an approximate estimation of the target sample size, informed by information power. We also noted in the protocol that, based on information power guidance, “in practice the appropriateness of the sample must be evaluated *during the research process.*” This is consistent with the information power approach to sample size specified in the protocol.^14^ We also note that this is consistent with the SPQR guidelines, which state “Although investigators often do not determine sample size a priori in qualitative research, they should nonetheless describe how they established the final sample size. When appropriate (e.g., when a flexible sampling strategy was used), they should explain the criteria used to decide when no further sampling was necessary”.^15^  We had added additional text to elaborate on the use of information power, as described in our response to the editor above.  Regarding concerns of the generalisability of findings and the stratification of finding according to demographics, we would like to note the following. Qualitative research is well suited to exploring meanings, developing theory, or hypothesis-generating. As such, qualitative research is not expected to be generalisable – and indeed this would not be possible with the sample sizes typical of qualitative research.^16^ However, we agree with the reviewer that this means the views of some key informants (particularly men, non-White individuals, and those with high school education) are not well-represented by this sample).  To address this comment, we have added the following text to the limitations:  “Second, although generalisability of findings is not a key aim of qualitative research, over-representation of particular demographic groups in the sample should be kept in mind when interpreting the experiences reported. Despite attempts to invite a broad range of participants, the sample was predominantly White, highly educated, women. Given that access to, familiarity with, and acceptability of mHealth may be impacted by gender, ethnicity, and education, future evaluations of PolarUs in more diverse populations is needed to explore any potential impacts of the digital divide on engagement.”  Regarding the use of purposive sampling and the risk of bias, we note that this is one of the most widely utilised approaches in qualitative research – it aims to select cases that are likely to be information rich with respect to this research aims. We note that the SPQR guidelines reference purposive sampling as an accepted way to satisfy Criteria 8: Sampling Strategy “Several sampling strategies are commonly used in qualitative research, although most fall under the umbrella of purposeful (or purposive) sampling. Purposeful sampling means that participants, documents, or events are selected for their relevance to the research question, based on guiding theory or experiences and assumptions of the researchers.”^15^  We have added the following text to the methods to further detail and justify the purposive sampling framework used:  “In line with SRQR standards, purposive sampling [29] was used to select participants to invite into the qualitative study”  “The approach to purposive sampling was theoretical; invited candidates were selected on the basis of diversity achieved in the interviewed sample to date.”  Finally, regarding the reviewer’s suggestion to stratify demographics: although it is common to describe individual demographic characteristics for transparency (i.e., ‘thick description’, an aspect of rigour), we have also added summary statistics to the results section:  “Sample characteristics are described in Table 1; 60% (n=15) were women, 92% (n=23) had a diagnosis of BD-I, 64% (n=16) reported White/European ethnicity, 56% (n=14) resided in Canada, 48% (n=12) were engaged in full time work, and the majority (88%; n=22) had completed some form of post-secondary education. The mean age of the sample was 38.3 years (*SD*=11.5).” |
|  | Thank you for introducing more details of the inductive approach used in thematic analysis (i.e., data were analyzed without preconceived theories). Although it increased the flexibility of identifying emerging themes and concepts from interviews, I still have some concerns that need further explanation. 1) The inductive approach relies heavily on the researchers’ interpretations, which can introduce bias and affect the credibility of the findings. Without a well-defined coding framework before starting the analysis, how did the authors determine which factors were related to their research questions and whether they should be extracted from the interview transcripts? If the authors did not provide categories, definitions, and examples of coding in advance, how did they ensure consistency among researchers? | In our previous response to this reviewer, we noted that our use of inductive coding meant that codes/themes generated are guided by participant responses, rather than pre-conceived theories or ideas. The inductive approach was selected for this study given it is capable or representing the unique contexts and experiences of participants, and it allows the research to remain open to new ideas that may not have been apparent when data is viewed through the lens of an existing framework. The inductive approach is explicitly contrasted with deductive approaches, in which pre-existing theories and framework are used to structure and interpret the data (e.g., developing a coding framework based on constructs specified in established models). This reviewer’s suggestion to have a defined coding framework in advance of the analysis would, *by definition*, not occur in an inductive approach to coding. As further elaborated by Braun and Clarke, thematic analysis is not a ‘one size fits all’ approach to methods, and these are vastly different approaches to coding that may be applied where justified by the aims of the specific qualitative paper.^17^  Although deductive coding has beneficial applications, such as being used to test theory or generate examples of how this theory can be grounded in a specific context, it is not universally applicable to all research aims. For example, a potential downside of this approach is that data that expands on or contradicts a theory may be missed. For this reason, in response to this reviewer’s concern, we previously elaborated on the rational for and the implications of the inductive approach in the previous iteration of the manuscript.  To ensure the credibility, confirmability, dependability, and transferability of the findings, we previously described in detail the use of several strategies to support rigour. We have added the following text to the methods to confirm that these strategies are in line with SRQR recommendations:  “Multiple techniques were used to support analytic rigor and trustworthiness, leveraging the widely-used criteria developed by Lincoln and Guba [66], as well as specific recommendations in the SRQR.” |
|  | 2) Although several researchers reviewed and discussed the codes and themes in thematic analysis, it appears that only one researcher (EM) initially read the transcript and performed the coding. To reduce the risk of bias, it is highly recommended that two or more researchers independently transcribe the interviews, read the transcripts, and perform the coding; and then they discuss to resolve any disagreements. | In our previous respect to this reviewer, we noted that noted that the concepts of reliability, validity, and generalisability assume a positivist paradigm (i.e., there is an objective reality, and it is only though the application of valid and reliable measurement tools and robust samples that we can make accurate inferences). These are not universally appropriate in qualitative research where different paradigms for knowledge generation are used. The quality of qualitative analysis is more typically evaluated using concepts such as trustworthiness and rigor. In this paper, we used multiple widely-accepted strategies to demonstrate rigor, including member checking and peer debriefing (which were commented on positively by Reviewers 1 & 2), and provided detailed examples of how our methodological decisions mapped onto these standards of credibility, transferability, dependability, and confirmability.  Accepted ways of demonstrating rigour in such papers published in PLOS Digital Health are in line with what was performed in this analysis, e.g., discussions during theme development with the research team, member checking. This approach is also in line with guidance provided by the SRQR (Item 15. Techniques to enhance trustworthiness and credibility of data analysis, e.g., member checking, triangulation, audit trail).^15^ We note again that the developers of thematic analysis Braun and Clarke have commented in depth on the problem in applying ‘coding reliability’ standards to their model to thematic analysis.^17^  To clarify our approach to thematic analysis and the quality practices that are most appropriate for this method, we added the following text to the methods section:  “In accordance with thematic analysis standards and to support reflexivity, we note that interviews and data analysis were conducted by the first author EM, a white woman, psychologist and mid-career researcher with expertise in QoL-focused interventions for BD and digital health intervention. Our use of a single primary analyst is in line with quality practices in reflexive thematic analysis; positivist ‘coding reliability’ approaches are inconsistent with the philosophy and values of this approach, where knowledge generation is situated as a contextual and interpretative process.”  This context is followed by a summary of strategies used to support rigor.  Although multiple coders may be appropriate in deductive qualitative approaches (e.g., content analysis that uses a pre-defined coding framework) our use of a single analyst is in line with multiple qualitative papers using thematic analysis published in PLOS Digital.^12,13,18-20^ |
|  | 3) Findings from inductive research are often context-specific, making it challenging to generalize results to broader populations or contexts. For example, the authors found drivers of engagement were shaped by contextual influences, including “involvement in a research study”; however, it is hard to use this finding to create robust and applicable insights for future practice. | In our response regarding the use of purposive sampling above, we described several position papers on the role of ‘generalisability’ in qualitative research,^16^ that are also relevant to this comment. Transferability, an aspect of rigour specific to qualitative research that describes the ability to apply findings in other settings, is supported in this work by the use of thick description of the research context and participant characteristics. This is described and referenced in the methods section.  Furthermore, the fact that findings are specific to the context of a research study offers important insights for interpreting the findings in future feasibility studies of mHealth interventions. As mentioned in our response to the editor above, the concern that the study environment positively biases engagement compared to real-world contexts has previously been raised.^2^ Our qualitative findings help illustrate the ways in which the trial environment may impact engagement, which has relevance for both the measurement and interpretation of quantitative indicators of app engagement.^1^  To acknowledge the fact that over-representation of particular demographics may mean the views of particular groups impacted by the digital divide may not be adequately represented, we added further comment to the limitations highlighting existing research about barriers to mHealth use faced by these groups, and the need for further research. This text is provided above in response to earlier comments about generalisability. |
|  |  | **Additional change:** Since the time of submission of this qualitative article, further cleaning and analysis of quantitative engagement statistics was undertaken for the primary outcomes paper. This has resulted in some minor changes to the daily and monthly adherence ratings described in the participant characteristics table. |

**References**

1. O'Brien HL, Morton E, Kampen A, Barnes SJ, Michalak EE. Beyond clicks and downloads: a call for a more comprehensive approach to measuring mobile-health app engagement. BJPsych Open. 2020;6(5):e86. Epub 2020/08/11. doi: 10.1192/bjo.2020.72.
2. Baumel A, Edan S, Kane JM. Is there a trial bias impacting user engagement with unguided e-mental health interventions? A systematic comparison of published reports and real-world usage of the same programs. Translational behavioral medicine. 2019;9(6):1020-33. doi: 10.1093/tbm/ibz147.
3. Bhandari A, Wagner T. Self-reported utilization of health care services: improving measurement and accuracy. Med Care Res Rev. 2006 Apr;63(2):217-35. doi: 10.1177/1077558705285298. PMID: 16595412.
4. Grodon J, Tack C, Eccott L, Cairns MC. Patient experience and barriers of using a mHealth exercise app in musculoskeletal (MSK) Physiotherapy. PLOS Digit Health. 2024 Oct 7;3(10):e0000626. doi: 10.1371/journal.pdig.0000626. PMID: 39374194; PMCID: PMC11458024.
5. Maliwichi P, Chigona W, Malata A. Factors motivating maternal healthcare clients to use mHealth interventions in rural Malawi. PLOS Digit Health. 2025 Apr 22;4(4):e0000805. doi: 10.1371/journal.pdig.0000805. PMID: 40261862; PMCID: PMC12013878.
6. Yelverton V, Gass SJ, Amoatika D, Cooke C, Ostermann J, Natafgi N, Hair NL, Olatosi B, Owens OL, Qiao S, Li X, Derrick C, Weissman S, Albrecht H. Telehealth or in-person HIV care? Qualitative study findings on decision-making from people with HIV and HIV care providers in South Carolina during the COVID-19 pandemic. PLOS Digit Health. 2025 Apr 8;4(4):e0000812. doi: 10.1371/journal.pdig.0000812. PMID: 40198619; PMCID: PMC11977962.
7. Blake HB, Njah M, Babey MM, Asongwe E, Junkins A, Dionne JA, Montgomery AE, Washington T, Ivankova N, Smith T, Jolly PE. Understanding female sex workers' acceptance of secret Facebook group for HIV prevention in Cameroon. PLOS Digit Health. 2024 Aug 14;3(8):e0000562. doi: 10.1371/journal.pdig.0000562. PMID: 39141653; PMCID: PMC11324143.
8. Braun, V., & Clarke, V. (2024). How do you solve a problem like COREQ? A critique of consolidated criteria for reporting qualitative research. *Methods in Psychology*, 100155.
9. Braun, V., & Clarke, V. (2019). To saturate or not to saturate? Questioning data saturation as a useful concept for thematic analysis and sample-size rationales. *Qualitative Research in Sport, Exercise and Health*, *13*(2), 201–216. <https://doi.org/10.1080/2159676X.2019.1704846>
10. Dossett LA, Kaji AH, Cochran A. SRQR and COREQ Reporting Guidelines for Qualitative Studies. JAMA Surg. 2021 Sep 1;156(9):875-876. doi: 10.1001/jamasurg.2021.0525. PMID: 33825809.
11. Cliffe B, Linton MJ, Haime Z, Biddle L. Young adults' experiences of using a young person's mental health peer support app: A qualitative interview study. PLOS Digit Health. 2024 Jul 31;3(7):e0000556. doi: 10.1371/journal.pdig.0000556. PMID: 39083458; PMCID: PMC11290682.
12. Healy D, Carr E, Conlan O, Browne AC, Walsh JC. Exploring the content of the STAND-VR intervention: A qualitative interview study. PLOS Digit Health. 2023 Mar 13;2(3):e0000210. doi: 10.1371/journal.pdig.0000210. PMID: 36913343; PMCID: PMC10010507.
13. Kearney M, Ryan L, Coyne R, Worlikar H, McCabe I, Doran J, Carr PJ, Pinder J, Coleman S, Connolly C, Walsh JC, O'Keeffe D. A qualitative exploration of participants' perspectives and experiences of novel digital health infrastructure to enhance patient care in remote communities within the Home Health Project. PLOS Digit Health. 2024 Nov 1;3(11):e0000600. doi: 10.1371/journal.pdig.0000600. PMID: 39485811; PMCID: PMC11530050.
14. Malterud K, Siersma VD, Guassora AD. Sample Size in Qualitative Interview Studies: Guided by Information Power. Qual Health Res. 2016 Nov;26(13):1753-1760. doi: 10.1177/1049732315617444. Epub 2016 Jul 10. PMID: 26613970.
15. O'Brien BC, Harris IB, Beckman TJ, Reed DA, Cook DA. Standards for reporting qualitative research: a synthesis of recommendations. Acad Med. 2014 Sep;89(9):1245-51. doi: 10.1097/ACM.0000000000000388. PMID: 24979285.
16. Varpio L, O’Brien B, Rees CE, et al. The applicability of generalisability and bias to health professions education’s research. Med Educ 2021; 55: 167–173.
17. Braun, V., & Clarke, V. (2020). One size fits all? What counts as quality practice in (reflexive) thematic analysis? *Qualitative Research in Psychology*, *18*(3), 328–352. https://doi.org/10.1080/14780887.2020.1769238
18. Tilley DO, McKeon B, Ibrahim N, Macdonald SH, Casey M. A snapshot on a journey from frustration to readiness-A qualitative pre-implementation exploration of readiness for technology adoption in Public Health Protection in Ireland. PLOS Digit Health. 2024 Mar 5;3(3):e0000453. doi: 10.1371/journal.pdig.0000453. PMID: 38442098; PMCID: PMC10914281.
19. Workman A, Campbell SL, Williamson GJ, Lucani C, Bowman DMJS, Cooling N, Johnston FH, Jones PJ. Understanding the perspectives and needs of multiple stakeholders: Identifying key elements of a digital health intervention to protect against environmental hazards. PLOS Digit Health. 2024 Jan 29;3(1):e0000444. doi: 10.1371/journal.pdig.0000444. PMID: 38285717; PMCID: PMC10824450.
20. Okpako T, Kale D, Perski O, Brown J. Developing content for a virtual reality scenario that motivates quit attempts in adult smokers: A focus group study with art-based methods. PLOS Digit Health. 2024 May 23;3(5):e0000512. doi: 10.1371/journal.pdig.0000512. PMID: 38781149; PMCID: PMC11115299.
